# Porcine Enteric Coronaviruses: Origins, Global Spread, Transmission Pathways and Pathogenesis

**DOI:** 10.1155/tbed/6796680

**Published:** 2026-09-02

**Authors:** Héctor Puente, Héctor Arguello, Pedro Rubio, Ana Carvajal

**Affiliations:** ^1^ Department of Animal Health, Faculty of Veterinary Medicine, Universidad de León, León, Spain, unileon.es; ^2^ INDEGSAL, Universidad de León, León, Spain, unileon.es; ^3^ IBIOLEÓN, Hospital de León, León, Spain, saludcastillayleon.es

**Keywords:** porcine deltacoronavirus (PDCoV), porcine epidemic diarrhoea virus (PEDV), swine acute diarrhoea syndrome coronavirus (SADS-CoV), swine enteric coronavirus (SeCoV), transmissible gastroenteritis virus (TGEV)

## Abstract

Porcine enteric coronaviruses (CoVs) constitute a persistent and evolving threat to global pig production due to their high transmissibility, environmental stability and capacity for rapid international dissemination. Historically, transmissible gastroenteritis virus (TGEV) and porcine epidemic diarrhoea virus (PEDV) have been the most significant enteric CoVs in swine, causing acute outbreaks with severe diarrhoea and high neonatal mortality. In the past decade, additional pathogens have expanded this epidemiological landscape of porcine enteric CoVs. Swine enteric CoV (SeCoV), porcine deltacoronavirus (PDCoV), and swine acute diarrhoea syndrome CoV (SADS‐CoV) illustrate the remarkable genetic plasticity, recombination capacity and interspecies transmission potential of this viral group. To address these challenges, this review provides a comprehensive synthesis of the main scope regarding the origins, global spread, transmission pathways, and pathogenesis of these viruses. We emphasise the critical differences among them, particularly concerning transboundary dissemination, host range, and zoonotic potential. In conclusion, the repeated emergence and host adaptation of these agents underscore the urgent need to address surveillance gaps through continuous molecular tracking, improved biosecurity protocols, and the development of coordinated global control strategies to mitigate their impact on animal health and modern production systems.

## 1. Introduction

Although systematic vaccination and improvements in management enable better control of porcine diarrhoea, legal restrictions on the use of antibiotics and zinc oxide in recent years have contributed to a greater impact of enteric disorders. Among them, viral diarrhoeas caused by coronaviruses (CoVs) have become progressively more challenging over the last 10–20 years due to the emergence and re‐emergence of new viruses and new variants with increased virulence and/or transmissibility [1–3].

Particularly, the occurrence and dissemination of highly virulent isolates of porcine epidemic diarrhoea virus (PEDV) in China and other Asian countries since 2010 [4, 5] as well as the emergence of this virus in the United States and other American countries in 2013 have highlighted the considerable potential of CoVs to emerge, spread rapidly, and cause severe economic losses in pig production and have focused increasing interest on this genus of enteric viruses.

Due to the importance of viral diarrhoea in pigs associated to CoVs, this review addresses the origin, global distribution, transmission pathways and pathogenesis of porcine enteric CoVs, with particular emphasis on transboundary spread, evolutionary dynamics and recombination events underlying their emergence. By integrating the state of the art on these viruses, we aim to identify common epidemiological patterns, key differences and relevant knowledge gaps for surveillance and control.

## 2. CoVs, Origins and Spread

**Figure 1:**
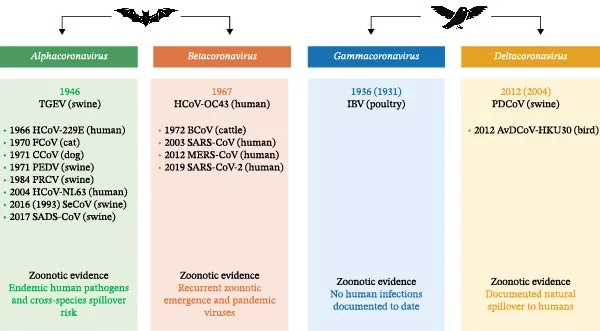
Taxonomic classification of coronaviruses (CoVs). Phylogenetic evidence indicates that alpha‐ and betacoronaviruses originated primarily from bat reservoirs, whereas gamma‐ and deltacoronaviruses likely emerged from avian hosts, with subsequent host jumps to other animals or humans. Representative viruses, year of first description, main host species and zoonotic evidence are summarised. The zoonotic status for each genus is categorised based on documented empirical data, reflecting recurrent pandemic emergence (*Betacoronavirus*), natural spillovers or endemicity (*Alpha*‐ and *Deltacoronavirus*) and the current absence of human infections (*Gammacoronavirus*). Porcine enteric CoVs include porcine epidemic diarrhoea virus (PEDV), transmissible gastroenteritis virus/porcine respiratory coronavirus (TGEV/PRCV), swine enteric coronavirus (SeCoV) and swine acute diarrhoea syndrome coronavirus (SADS‐CoV) within the *Alphacoronavirus* genus, as well as porcine deltacoronavirus (PDCoV) within the *Deltacoronavirus* genus. Other representative CoVs from different host species are shown, including human respiratory coronavirus: HCoV‐229E, HCoV‐OC43, HCoV‐NL63, severe acute respiratory syndrome coronavirus (SARS‐CoV), middle east respiratory syndrome coronavirus (MERS‐CoV), severe acute respiratory syndrome coronavirus ‐ 2 (SARS‐CoV‐2), infectious bronchitis virus (IBV), canine coronavirus (CCoV) and feline coronavirus (FCoV).

CoVs belong to the family *Coronaviridae* (order *Nidovirales*) and are currently classified into four genera: *Alphacoronavirus*, *Betacoronavirus*, *Gammacoronavirus*, and *Deltacoronavirus*. Phylogenetic evidence suggests that alpha‐ and betacoronaviruses originated primarily from bat reservoirs, whereas gamma‐ and deltacoronaviruses likely emerged from avian hosts, with subsequent host jumps by a high mutation and recombination rate that confers remarkable adaptability (Figure 1). This evolutionary plasticity allows CoVs to consistently breach species barriers and adapt to novel hosts [3, 6, 7].

Historically, CoVs were recognised as significant animal pathogens long before their identification in humans. The first CoV identified was avian infectious bronchitis virus in 1936, followed by descriptions in mice, cats, dogs, and pigs throughout the mid‐20th century [8]. In humans, while early alphacoronaviruses (Human coronavirus 229E) and betacoronaviruses (human CoV OC43) identified in the 1960s were associated only with the common cold, the emergence of severe acute respiratory syndrome CoV (SARS‐CoV), Middle East respiratory syndrome CoV (MERS‐CoV), and SARS‐CoV‐2 highlighted the severe zoonotic potential and public health risk of this viral family [9].

In swine, several enteric CoVs of veterinary importance have emerged across these phylogenetically distinct lineages (Table 1). PEDV and transmissible gastroenteritis virus (TGEV) are classical *Alphacoronavirus*, long recognised as major causes of acute diarrhoea outbreaks and high mortality in suckling piglets. More recently, the swine acute diarrhoea syndrome CoV (SADS‐CoV), another *Alphacoronavirus*, has been associated with devastating outbreaks of neonatal diarrhoea in Asia, while porcine deltacoronavirus (PDCoV) has emerged as a novel enteric agent in Asia and America. Finally, the emergence of swine enteric CoV (SeCoV) in Europe—a chimeric virus generated by recombination between TGEV and PEDV—exemplifies how this genetic plasticity continues to drive the diversification of swine pathogens [18, 22, 38, 39].

**Table 1:** Origin, distribution, host range and virulence of porcine enteric coronaviruses.

Viral agent	First report and distribution	Proposed origin	Replication in other species	Pathogenicity for other species	References
*Alphacoronavirus porci* PEDV	1971 (United Kingdom). Global distribution (America, Asia and Europe)	Spillover and evolutionary divergence from an ancestral bat coronavirus	In vitro: African green monkey kidney cells (Vero). Natural/Experimental: not established in vivo	Not reported	[3, 4, 10, 11]
*Alphacoronavirus suis* TGEV	1946 (United States). Global distribution (Africa, America, Asia and Europe)	Spillover and evolutionary divergence from a common ancestor shared with canine/feline coronaviruses	Natural and experimental: dogs, cats and foxes	Dogs, cats, foxes: strictly subclinical/asymptomatic infection (viral shedding and seroconversion without clinical disease)	[3, 12–14]
*Alphacoronavirus suis* PRCV	1984 (Belgium). Global distribution (Europe, North America and Asia)	Natural deletion mutant of TGEV (spontaneous large deletion in the Spike gene)	Not reported	Not reported	[13, 15–17]
SeCoV	2016 (Italy) (retrospectively detected 1993 in Spain). Europe (Spain, Italy, Germany and Slovakia)	Recombination event (TGEV/PRCV genetic backbone incorporating a PEDV Spike gene)	Not reported	Not reported	[18–21]
*Deltacoronavirus suis* PDCoV	2012 in Hong Kong (retrospectively detected in mainland China in 2004; widespread pathogenic emergence in the United States in 2014) America (United States, Canada, Mexico and Peru) and Asia (China, South Korea, Japan, Thailand, Vietnam, Laos and Taiwan)	Inter‐class host jump facilitated by a recombination event between two ancestral avian deltacoronaviruses	Natural: humans (children) Experimental: calves, chickens, turkey poults, mice and ferrets In vitro: broad range (human, bovine, avian, feline and canine cell lines)	Humans: mild acute undifferentiated febrile illness Poultry (chickens/turkeys) and calves: experimental acute enteric disease (diarrhoea and intestinal lesions) Ferrets: subclinical to mild experimental infection (viral shedding and seroconversion without severe clinical disease)	[22–29]
SADS‐CoV	2017 (China). Asia (China and Vietnam)	Direct trans‐species spillover from Rhinolophus bat coronaviruses (HKU2‐related strains)	Natural: not reported Experimental: mice (suckling and adults) and avian species (chickens) In vitro: exceptionally broad range (human respiratory/intestinal, bat, rodent, chicken, feline and canine cell lines)	Suckling mice: experimental severe enteric disease with acute watery diarrhoea and high mortality Adult mice: subclinical infection (targeted replication in splenic dendritic cells) Poultry (chickens): subclinical infection (viral shedding without overt clinical signs or lesions)	[30–37]

Abbreviations: PDCoV, porcine deltacoronavirus; PEDV, porcine epidemic diarrhoea virus; PRCV, porcine respiratory coronavirus; SADS‐CoV, swine acute diarrhoea syndrome coronavirus; SeCoV, swine enteric coronavirus; TGEV, transmissible gastroenteritis virus.

## 3. PEDV

### 3.1. Origin and Global Distribution

Porcine epidemic diarrhoea (PED) is a highly contagious infectious disease caused by an *Alphacoronavirus* of the subgenus *Pedacovirus*, designated as *Alphacoronavirus porci* by the International Committee on Taxonomy of Viruses (ICTV) in 2023 and formerly named as PEDV. It causes a disease characterised by watery diarrhoea and vomiting in pigs of all ages, although the most severe disease occurs in neonatal piglets during the first week of life, with intense dehydration, which may result in mortality rates approaching 100% in severely affected litters [3, 40].

The disease was first described in the United Kingdom and Belgium in the early 1970s, although the origin of the virus in domestic pigs remains uncertain [41, 42]. Recent phylogenetic studies have shown a close relationship between PEDV and a CoV detected in insectivorous bats (*Tadarida brasiliensis*) in Brazil, supporting a direct bat‐to‐pig host jump as the initial event in PEDV emergence [10].

Following its identification, PEDV spread rapidly during the 1980s to all countries with significant pig production in Europe, causing epidemic outbreaks characterised by seasonal presentation, usually in colder months, with high morbidity (80%–100%) and moderate to high mortality in lactating piglets (20%–50%) [3, 41, 43]. Endemic or enzootic presentations with lower morbidity and mortality rates were sometimes developed in PEDV‐infected farms with diarrhoea mainly observed in weaned piglets, when passive maternal immunity wanes, or in young seronegative replacement sows [44].

Throughout the 1990s, relevance of PEDV in Europe decreased progressively, and PED evolved into a sporadic disease in European pig production, with occasional epidemics as it occurred in northern Italy in 2005–2006, affecting over 50 farms in a high‐density pig region [45].

In contrast, after its first description in China and Japan in 1983, PEDV has remained as a major cause of diarrhoea on pig farms in Asia. The widespread use of inactivated and attenuated vaccines in the region seems to have favoured virus persistence through the emergence of new variants [44, 46]. From 2010 onwards, particularly severe PED outbreaks occurred in southern China, with 50%–90% mortality in neonatal piglets, rapidly spreading across the country and Southeast Asia. These outbreaks were attributed to the emergence of highly virulent PEDV strains [44, 47].

The introduction of PEDV to the Americas in 2013 marked a turning point in the global epidemiology of this CoV. The first affected farm was identified in Iowa in April 2013 and confirmed in May. The virus explosively spread across the United States, affecting over 5500 farms in 25 states within the first year and extending to Canada, Mexico, Peru, the Dominican Republic, Colombia, and Ecuador [48, 49]. Over 50% of United States pig farms were infected within 2 years since the first description, with animal and personnel movements, transport trucks, and contaminated feed as key factors in this rapid spread. Gradually, outbreaks declined in both frequency and severity, likely due to the development of specific immunity. Phylogenetic analyses revealed that American PEDV isolates were closely related to Asian strains circulating in preceding years. Source tracking analyses pointed flexible containers used in international raw material transport as the most probable source for viral introduction into the continent [4, 50].

The American epidemic had global consequences; PEDV re‐emerged in Asian countries such as Japan or South Korea, possibly due to trade with the United States [15, 51], as well as in several European countries during the winter 2013–2014 [52–54]. Despite low pre‐existing immunity, both clinical impact and virus spread were limited in Europe, contrasting with the American experience [5, 41].

**Figure 2:**
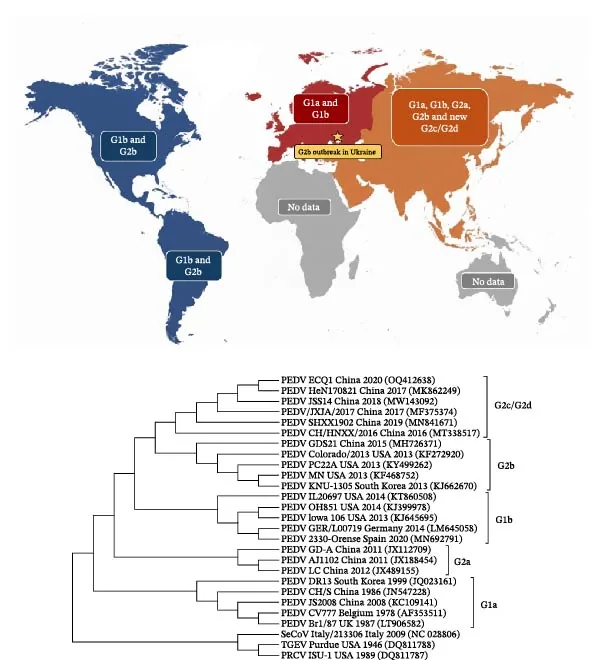
Global distribution and phylogenetic relationships of porcine epidemic diarrhoea virus (PEDV) genogroups. The map shows the global distribution of PEDV genogroups based on reported detections at the continental level. Classic INDEL strains (G1a) have been reported in Europe and Asia between 1971 and 2010, whereas new INDEL strains (G1b) have been detected in Asia, America and Europe. Non‐INDEL strains belonging to genogroup G2a are restricted to Asia, while new non‐INDEL strains (G2b) have been reported in Asia as well as in America from 2013 onwards. This distribution implies that international trade logistics act as primary drivers for the transboundary introduction of virulent G2b strains. Additional subgenotypes G2c and G2d have been described exclusively in Asia. A single outbreak caused by a G2b strain was confirmed in Europe, in Ukraine in 2014, and is highlighted in the figure. Africa and Oceania are shown in grey, indicating the absence of reported data. The phylogenetic tree based on complete genome sequences shows representative PEDV strains from each genogroup, including information on the country and year of detection. The tree was constructed using the Neighbour‐Joining method and also includes sequences of transmissible gastroenteritis virus (TGEV), porcine respiratory coronavirus (PRCV), and swine enteric coronavirus (SeCoV) as outgroups. The tree topology illustrates the overall evolutionary divergence that separates historical classical strains from contemporary global outbreak genogroups.

The availability of high‐throughput sequencing techniques together with the high number of affected farms enabled significant advances in PEDV molecular characterisation. Currently, more than 400 complete genomes are available in GenBank. Although overall nucleotide identity is high (>90%–95%), highly variable regions have been identified, particularly in the spike protein or S‐protein gene [55]. Based on the sequence of this region, two major groups are distinguished: the INDEL or G1 isolates, with insertions and deletions in S1, associated with milder outbreaks, and the non‐INDEL or G2 isolates, generally more virulent (Figure 2). Experimental studies have confirmed the differences in virulence and transmissibility between INDEL or G1 and non‐INDEL or G2 isolates [4, 56, 57].

As illustrated in Figure 2, the global molecular epidemiology of PEDV exhibits marked geographic compartmentalisation across its distinct subclusters:

• G1a (classical strains): this cluster includes historical prototype strains from the 1970s and 1980s (such as CV777 and Br1/87) alongside early Asian isolates. Today, they primarily serve as reference and vaccine strains rather than dominant field‐circulating viruses [4, 42, 46].
• G1b (S‐INDEL strains): comprising more recent isolates, this subgroup is globally distributed across Europe, the Americas, and Asia, representing the main cause of mild, sporadic PEDV outbreaks in Western and Central Europe [5, 42, 49].
• G2a (early pandemic strains): this subgroup includes the highly virulent strains responsible for severe epidemic waves in China between 2010 and 2012, with circulation remaining largely restricted to Asia [4, 43].
• G2b (global pandemic strains): representing the highly virulent isolates responsible for the 2013 epidemic in the United States, G2b strains are widely established across the Americas and parts of Asia [1, 48]. Crucially, mainland Europe remains free of G2b, with the single exception of an isolated outbreak in Ukraine in 2014 [58].
• G2c and G2d (emerging variants): in recent years, ongoing viral evolution, immune pressure from widespread vaccination, and recombination events in endemic settings have driven the emergence of these new subgroups in Asia [59, 60].

Consequently, the epidemiological landscape of PEDV differs markedly across continents. While the Americas experience the co‐circulation of G1b and highly virulent G2b strains, Europe maintains a more favourable situation restricted almost exclusively to G1a and G1b. In contrast, Asia represents the most complex epidemiological scenario, serving as an evolutionary melting pot where all known subgroups (G1a, G1b, G2a, G2b, G2c and G2d) currently co‐circulate.

### 3.2. Sources of Infection and Transmission Pathways

PEDV is transmitted primarily via the faecal–oral route, both directly between animals and indirectly through the environment. Infected pigs shed large amounts of virus in faeces from the first or second day post‐infection onwards, and susceptible animals are infected by the ingestion of contaminated material [3, 15]. Transmission efficiency is enhanced by several factors: high viral shedding in faeces [61–65], prolonged excretion periods—which can reach 3–6 weeks and extend beyond clinical resolution, resulting in convalescent carriers [57, 62, 66]—and the low infectious dose required to initiate infection [67].

PEDV also exhibits sufficient environmental stability to allow indirect transmission. The virus can remain viable in faeces or slurry for up to 2 weeks at 25°C and up to 28 days at 4°C and survives in dry and wet feed for several days [68]. These features facilitate dissemination via water, feed and fomites such as trucks, clothing or footwear, particularly under cold and humid conditions [69, 70]. As an enveloped virus, PEDV remains sensitive to detergents and most standard disinfectants.

During the American epidemic, other potential transmission routes were thoroughly investigated. Although viral RNA was detected in air samples close to infected farms [71], no infectious virus was demonstrated in sufficient quantities. Similarly, viral material has been found in milk and semen but without evidence of infectivity [72]. Transmission via spray‐dried porcine plasma was excluded as the virus was inactivated during processing and storage [73, 74]. Replication in other species besides pigs has not been demonstrated, although mechanical transmission via vectors cannot be ruled out [75]. An alternative pathway of airborne PEDV dissemination linked to transient nasal epithelium replication and dissemination to the intestinal mucosa using dendritic cells and T cells has been recently proposed and would play a role in PEDV dissemination under certain conditions [76]. Finally, transplacental transmission has been suggested under field conditions as viable PEDV was isolated from umbilical cords of piglets born to PEDV‐positive, clinically asymptomatic sows; phylogenetic analysis indicated that the detected viruses clustered with G1a strains [77].

## 4. TGEV

### 4.1. Origin and Global Distribution

Transmissible gastroenteritis (TGE) is, like PED, a highly contagious enteric disease caused by an *Alphacoronavirus* of the subgenus *Tegacovirus*, also recently nominated by ICTV *Alphacoronavirus suis*. Clinically, it is characterised by vomiting, severe watery diarrhoea, and high mortality in piglets under 2 weeks of age, reaching 100%, making it one of the most devastating viral enteric diseases in pig production [3, 38, 40].

TGE was first described in the United States in 1946 [12] and subsequently spread to almost all countries with intensive pig production. In the 1960s and 1970s, TGEV was a major cause of piglet mortality worldwide, with winter epidemic outbreaks similar to those later described for PEDV [3]. However, global epidemiology changed radically from the mid‐1980s due to the emergence of porcine respiratory coronavirus (PRCV) [13].

PRCV is a natural mutant of TGEV, with a deletion of ~200 amino acids at the N‐terminal of the S‐protein, profoundly altering the tropism from the gastrointestinal to the respiratory tract [16]. This deletion also prevents binding to sialic acid, a key step for interaction with intestinal mucin, resulting in the loss of enteropathogenicity [78].

PRCV was first detected in Europe in 1984 when high seropositivity to TGEV was observed without compatible clinical signs [15]. Its isolation in 1986 confirmed the existence of a closely related respiratory mutant [17]. Rapid airborne dissemination in the European pig population, and later in North America, induced effective cross‐immunity to TGEV, markedly reducing TGE incidence [3, 13].

Currently, TGE is sporadic in Europe and North America, primarily affecting PRCV‐seronegative farms. In Asia, TGEV continues to circulate alongside PEDV, causing severe diarrhoeal outbreaks in piglets and requiring differential diagnosis between both viruses [3]. Unlike PED, TGE is a notifiable disease to the World Organisation for Animal Health, reflecting its potential health and economic impact [13].

From an evolutionary perspective, TGEV and PRCV cluster with canine and feline CoVs within the subgenus *Tegacovirus* of *Alphacoronavirus*. In fact, TGEV is proposed to have originated from a canine CoV type II via a host jump, based on the ORF3 sequence [14]. This interspecies origin highlights the key role of host jump events in porcine CoV emergence. The identification of a recombinant virus between TGEV and canine CoV (subtype IIb) in dogs further demonstrates the high genetic plasticity of this group of viruses [79]. Moreover, recent studies have reported additional recombinant events involving TGEV. Besides the classical chimeric SeCoV derived from TGEV and PEDV, recombinations between TGEV and SADS‐CoV have also been described, indicating that TGEV can participate in multiple interspecies recombination events [80].

### 4.2. Sources of Infection and Transmission Pathways

TGEV‐infected pigs shed the virus mainly in faeces. Unlike PEDV, TGEV may persist in the intestinal tract or faeces for exceptionally long periods, over 100 days or even 18 months, although without conclusive evidence of persistent shedding of viable virus. Moreover, respiratory secretions and milk can also be infectious [81–83].

Direct transmission occurs via faecal–oral and also through the short‐distance aerosol route (<1 m), reflecting dual enteric and respiratory tropism. Indirect transmission is epidemiologically important and occurs via feed, water, trucks, clothing, footwear, and other contaminated fomites, particularly at low temperatures, which favour viral environmental survival [3, 44].

TGEV has a broader host range than PEDV; dogs, cats, and foxes can be infected and shed the virus for 1–2 weeks without clinical signs, potentially acting as sources of infection. Birds and insects may also serve as mechanical vectors, contributing to farm‐to‐farm spread [15, 44].

## 5. SeCoV

### 5.1. Origin and Global Distribution

**Figure 3:**
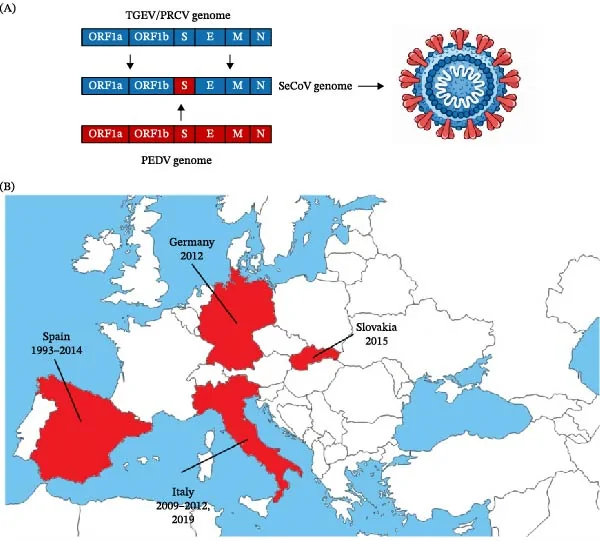
(A) Schematic representation of the genetic recombination event leading to the emergence of chimeric swine enteric coronavirus (SeCoV) between the transmissible gastroenteritis virus/porcine respiratory coronavirus (TGEV/PRCV) genome (blue) and the porcine epidemic diarrhoea virus (PEDV) genome (red). This alignment demonstrates a precise double‐recombination mechanism. The resulting SeCoV genome retains the TGEV/PRCV genetic backbone—comprising the ORF1a and ORF1b polyproteins, as well as the E, M and N structural genes—while incorporating the PEDV‐derived Spike (S) gene. The bottom right panel displays the SeCoV virion model, highlighting the PEDV‐derived spike proteins (red) assembled onto the TGEV‐based viral scaffold (blue), showing how structural chimerism allows the virus to maintain TGEV replication kinetics while adopting PEDV antigenicity to escape pre‐existing TGEV/PRCV‐specific herd immunity. (B) Geographical distribution of swine enteric coronavirus (SeCoV) in Europe. Countries where SeCoV has been detected are identified in red, with year of first identification. Geographical distribution and identification timeline suggests that SeCoV maintains a more restricted and slow‐spreading endemic pattern in Europe compared to its parental strains.

This chimeric virus was generated by recombination between TGEV or its respiratory mutant, PRCV, and PEDV; its genome is mostly derived from TGEV, but the gene encoding the S‐protein is replaced by that of PEDV (Figure 3A). This unique genetic structure represents a diagnostic challenge as detection depends on the molecular or serological targets used [18, 19]. Tests targeting the S protein or gene will misidentify SeCoV as PEDV, while detection of N or M proteins or genes will indicate TGEV infection. Reliable SeCoV detection requires combining assays targeting both viruses and different genomic regions, including PEDV S and TGEV N or M genes [18].

To date, this recombinant virus classified in the same viral species as TGEV, *Alphacorovirus suis*, has only been reported in Europe (Figure 3B). The first identification was in Italy in 2016, retrospectively analysing samples from enteric disease outbreaks between 2009 and 2012 [19]. Subsequent detections occurred in Germany in 2012 [20] and Slovakia in 2015 [21, 84]. A retrospective study by our research group showed that SeCoV circulated in Spain between 1993 and 2014, causing outbreaks misdiagnosed as PED [18]. The virus was detected again in Italy in 2019 [85], although subsequent studies have not demonstrated active circulation in Europe [70, 86].

This chimeric virus necessarily arose from the co‐infection of a single porcine cell by TGEV/PRCV and PEDV, enabling genetic recombination. Available evidence suggests that this event likely occurred in Europe, in a context of significant co‐circulation of both parental viruses [13].

### 5.2. Sources of Infection and Transmission Pathways

Epidemiological data on SeCoV are very limited. A study in Slovakia in 2015 documented its introduction via two breeding boars from Norway [21]. These animals exhibited compatible signs of infectious gastrointestinal disease, including anorexia and diarrhoea. The infection spread rapidly within the farm, likely via the faecal–oral route and through staff contact (boots and clothing). Gestating sows were the first animals affected, followed by all piglets in the three farrowing rooms. The infection then spread rapidly among fattening pigs, affecting the entire farm within 2 weeks. Within 3–4 weeks, the outbreak extended to a nearby farm with a different owner, located just 100 m away, showing a similar infection dynamic.

At present, it is unknown whether the transmission mechanisms and host range of this chimeric virus are more similar to TGEV or, on the contrary, to those of PEDV.

## 6. PDCoV

### 6.1. Origin and Global Distribution

An emerging enteric CoV affecting swine, PDCoV or recently named by ICTV *Deltacoronavirus suis*, is associated with acute diarrhoea, vomiting, dehydration and mortality in neonatal piglets. Its recognition as a pathogen in pigs is relatively recent and closely linked to the emergence of PEDV in North America [3, 40, 87]. This emergent CoV, a *Deltacoronavirus* within the *Buldecovirus* subgenus, was first described in 2012 in porcine faecal samples from clinically healthy pigs in Hong Kong [23]. Later, in January 2014, during a PEDV outbreak in the United States, molecular techniques applied to diarrhoeal samples detected this virus in some of the affected pigs [24]. Subsequent experimental studies confirmed its pathogenicity, causing a clinical disease in piglets indistinguishable from that associated with PEDV or TGEV infection [88].

The exact time and route of PDCoV introduction in the United States are unclear, but retrospective serological studies have demonstrated its circulation from at least 2010, remaining undetected for several years [89]. Similarly, retrospective analyses have shown circulation in China since 2004 [90]. Currently, PDCoV has been detected in numerous countries in America and Asia, including Hong Kong, the United States, Canada, China, South Korea, Japan, Thailand, Vietnam, Laos, Taiwan, Mexico, Haiti and Peru [25, 91–93]. Notably, PDCoV has not been reported in Europe to date [25, 70].

Phylogenetic analyses suggest that PDCoV likely originated in Southeast Asia in the 1990s [94], probably as a consequence of one or more recombination events involving avian deltacoronaviruses from parrots (HKU17 or ISU73347 CoVs) and quails and partridges (HKU30 CoV) [25]. Related deltacoronaviruses have been identified in wild mammals, such as mustelids and felids, suggesting a complex ecology with the potential participation of intermediate hosts [23, 26, 95].

### 6.2. Sources of Infection and Transmission Pathways

The primary route of transmission of PDCoV is faecal–oral, similar to that described for PEDV and TGEV. Faeces, vomitus and contaminated fomites are the main sources of infection. Viral RNA shedding in faeces after experimental infection has been detected for 19–28 days, exceeding clinical disease [96, 97]. Convalescent carrier animals may participate in within farm and farm‐to‐farm spread, as described for PEDV and TGEV. However, faecal viral load is lower than in other porcine enteric CoVs, and within farm spread is slower, suggesting suboptimal adaptation to pigs [98].

This CoV has a broad host range, infecting poultry such as chickens and turkeys, as well as domestic and laboratory mammals, including cattle, rodents, and ferrets [25–28, 99]. It has also been detected in plasma samples from Haitian children with febrile illness and respiratory or enteric signs, indicating potential human infection [29]. This broad tropism is linked to aminopeptidase N (APN) as the primary viral receptor, a highly conserved molecule across species. Hence, in vitro infection via APN has been demonstrated for PDCoV in porcine, human, and avian cells [100, 101].

## 7. SADS‐CoV

### 7.1. Origin and Global Distribution

The recently described SADS‐CoV is an *Alphacoronavirus*, first detected in 2017 during a devastating enteric disease outbreak in southern China, causing ~24,500 piglet deaths and substantial economic losses [30–32]. The virus re‐emerged in January 2019 in Guangdong province; retrospective studies showed circulation since at least August 2016 [102]. Although the first reports of SADS‐CoV were limited to Guangdong and Fujian provinces in China, subsequent seroepidemiological studies have indicated a wider distribution within the country [33]. Notably, a comprehensive nationwide survey analysing 12,978 sera from 29 Chinese provinces reported an unexpectedly high seropositivity rate of 59.97% [103]. However, these findings must be interpreted with critical caution. As acknowledged by the authors, there is potential for serological cross‐reactivity with an as‐yet‐unidentified pathogen. Therefore, this high seroprevalence highlights the urgent need for highly specific differential diagnostics rather than definitively confirming widespread SADS‐CoV infection. In recent years, the epidemiological footprint of SADS‐CoV has continued to expand, with documented re‐emergence in Guangxi province in 2021 and subsequent detection in inland regions such as Henan province in 2023 [104] and confirmed positive cases in Jiangxi province [34].

Epidemiologically, SADS‐CoV outbreaks display a localised and sporadic pattern without discernible temporal continuity. In a broad surveillance study across southern China (2021–2023), SADS‐CoV was detected in 5.16% (11/213) of investigated pig farms [34]. Genomic and phylogenetic analyses of these recent strains demonstrate a high overall nucleotide identity (~98.4%–98.8%) with earlier isolates; however, they exhibit discrete sequence variations and minor recombination events within the highly variable spike (S) gene, indicating continuous intra‐host adaptation and viral evolution within swine populations [104]. Moreover, retrospective analysis of porcine faecal samples from Vietnam has detected SADS‐CoV by RT‐qPCR in 5 out of 69 tested samples with high sequence homology to Chinese strains, representing the first and only report of this virus outside China to date [34, 35, 105].

Genomic and phylogenetic analyses have demonstrated high similarity (>95%) between SADS‐CoV and bat CoV HKU2, an *Alphacoronavirus* of subgenus *Rhinacovirus* isolated from horseshoe bats (*Rhinolophus ferrumequinum*) in Hong Kong and Guangdong between 2013 and 2016 [30, 31, 33], supporting direct bat‐to‐pig transmission, analogous to other emergent CoVs. Moreover, as we have already mentioned, recombination between TGEV and SADS‐CoV has been reported [80].

### 7.2. Sources of Infection and Transmission Pathways

Like other porcine enteric CoVs, SADS‐CoV is presumably transmitted via the faecal–oral route, with infected pig faeces as the main source. Although specific data are limited, the epidemiological mechanisms are likely similar to PEDV and TGEV [1, 87]. Regarding alternative transmission routes, experimental evidence is very limited, and the potential airborne or vector‐borne transmission has not been fully documented. However, experimental cohabitation studies demonstrate rapid transmission to direct‐contact piglets, with severe diarrhoea developing rapidly [34]. Drawing parallels from the transmission dynamics of other enteric CoVs, dissemination via infectious aerosols over short distances or indirect transmission through vehicles, such as food, water or different fomites, and vectors cannot be entirely ruled out and warrants further systematic investigation [71, 75, 76].

Notably, SADS‐CoV may infect multiple species [36, 37]. The S‐protein contains multiple insertions and deletions compared with bat HKU2, suggesting adaptation to new hosts [31, 106]. SADS‐CoV replicates efficiently in vitro using cell lines from various species, including human cells, raising questions about its potential broad host range [107–109]. Consistent with this observation, the cellular receptor for SADS‐CoV has not yet been identified, and the virus does not appear to utilise several well‐known CoV receptors such as APN, angiotensin‐converting enzyme 2 (ACE2), or dipeptidyl peptidase 4 (DPP4), suggesting that viral entry may rely on a more conserved host factor that could contribute to its broad cell tropism and cross‐species infectivity [110]. However, recent primary evidence has elucidated critical aspects of the entry mechanisms. It has been demonstrated that the S1^B^ region of the spike protein contains the principal receptor‐binding site. Furthermore, viral attachment and cellular entry are actively facilitated by host factors, including sialic acid and heparan sulphate, alongside proteolytic activation by furin cleavage and type II transmembrane serine protease (TMPRSS2) [111]. This complex network of host factors and cofactors mediates cellular attachment and membrane fusion, contributing to its broad cell tropism and cross‐species infectivity [110].

## 8. Pathogenesis, Clinical Presentation and Distinctive Virulence Patterns

Despite their genetic diversity, all porcine enteric CoVs share a remarkably similar baseline pathogenicity and clinical presentation. Following oral infection, these viruses target the small intestine, preferentially infecting and multiplying within mature enterocytes on the villi of the jejunum and ileum. Viral replication in the cytoplasm leads to enterocyte necrosis and desquamation, resulting in marked villous atrophy and, in some cases, destruction of capillaries and the central lacteal [3, 112–114]. Functionally, this widespread enterocyte destruction causes severe malabsorption and digestive dysfunction, culminating in an osmotic diarrhoea that is exacerbated by undigested nutrients in the intestinal lumen [4, 15]. Macroscopically, infections are characterised by the thinning of the intestinal wall, accumulation of yellowish fluid and gas in the lumen, and stomachs distended with curdled milk [3, 22, 31, 32, 87, 88, 98].

Clinically, infections are characterised by an acute onset of profuse watery diarrhoea, frequent vomiting, anorexia, dehydration, weight loss, lethargy, and occasionally fever, although SADS‐CoV infection typically presents without fever [34]. A critical shared feature across all these CoVs is their strong age‐dependent severity. Neonatal and suckling piglets (particularly under 1 week of age) exhibit the most severe disease, with mortality often approaching 80%–100% [1, 6, 38, 41, 45, 48, 115]. This extreme vulnerability is largely attributed to the slower enterocyte turnover in neonates compared to older animals [3]. Conversely, in weaned pigs and adults, the disease is generally milder and self‐limiting; although morbidity remains high, mortality rarely exceeds 1%–3% [15, 38, 47, 87, 114].

Beyond these common pathophysiological mechanisms, important differences exist among these viruses regarding receptor usage, extraintestinal tropism, and relative virulence. In the case of PEDV, intestinal tropism is largely determined by interactions between the viral spike (S) protein and multiple host cell surface molecules. Porcine APN (pAPN/CD13) has been proposed as an entry receptor, while sialic acid and heparan sulphate can act as attachment factors; however, accumulating evidence suggests that PEDV entry involves several receptors and host cofactors rather than a single essential receptor [110, 116]. Regarding extraintestinal tropism, PEDV replication in alveolar macrophages has been demonstrated in vitro but not in PEDV‐infected pigs [117], while the epithelium lining the nasal cavity could support viral replication after intranasal inoculation [76]. It remains unclear whether the transient detection of viral RNA in plasma samples is a true viremia or simply absorption of RNA from the intestinal lumen [3, 42]. Furthermore, PEDV has exceptionally been detected in reproductive tissues [77] with unclear clinical significance, and occasional reproductive disorders in sows are likely related to feedback practices rather than direct viral effects [118, 119]. For TGEV, viral entry is strongly mediated by pAPN/CD13, which is highly expressed on the apical surface of differentiated enterocytes, largely determining their intestinal tropism, while sialic acids act as attachment factors [11, 110, 120]. Unlike PEDV, TGEV extraintestinal replication is well documented, with multiplication occurring in alveolar macrophages and the mammary tissue [81]. Oronasal infection may induce pneumonia [83], and replication in mammary glands may relate to agalactia in infected sows, worsening litter mortality [3]. The TGEV‐derived PRCV replicates almost exclusively in the respiratory tract and rarely causes clinical signs, but it has major epidemiological importance by inducing cross‐protection against TGEV, though not against other CoVs [13, 15]. Like PEDV, TGEV presents both epidemic and endemic epidemiological patterns [13, 87, 113, 121].

Regarding recombinant and more recently emerged viruses, the impact of recombination on SeCoV biology remains largely unknown. A single experimental study comparing SeCoV and PEDV infection in weaned piglets reported no significant differences in clinical signs, suggesting similar virulence in this model [65, 122]. However, further studies are needed to evaluate extraintestinal tropism and cellular receptors, a task currently hampered because SeCoV has not been adapted to in vitro growth in cell culture using PEDV or TGEV protocols. Under field conditions on affected farms, SeCoV mortality is restricted to suckling piglets, ranging from 30% to 35% [21]. On the other hand, unlike the alphacoronaviruses, replication of PDCoV occurs not only in the small intestine but also, to a lesser extent, in the large intestine. Clinical signs and lesions are nearly indistinguishable from PEDV or TGEV but typically milder, consistent with a lower overall virulence [22, 88, 98]. In breeding farms, PDCoV infection is often self‐limiting due to lactogenic immunity, and mortality is particularly lower when it acts as a single pathogen, though coinfections with PEDV or TGEV are common [88, 98]. Finally, while producing similar enteric lesions, SADS‐CoV displays unique pathogenic features. In piglets under 5 days of age, clinical disease progresses rapidly, leading to severe dehydration and death within 2–6 days post‐infection [34]. Recent studies have shown that SADS‐CoV can infect suckling mice, causing severe clinical signs and mortality, thereby providing a unique model to investigate age‐dependent severity and host immune responses [36]. Additionally, in adult mouse models (C57BL/6J), SADS‐CoV produces subclinical infection with targeted viral replication occurring in splenic dendritic cells [34]. Furthermore, although primarily detected in the intestine, SADS‐CoV RNA has also been identified in the heart, liver, spleen, kidneys, stomach and lungs, suggesting a limited systemic dissemination during acute infection [115].

## 9. Comparative Epidemiological and Pathogenic Patterns Across Porcine Enteric CoVs

### 9.1. Determinants of Transboundary and Global Spread

Porcine enteric CoVs exhibit variable capacities and mechanisms for transboundary dissemination, dictated by a complex interplay of viral stability, host shedding dynamics, evidence strength across transmission routes and swine industry logistics. While both PEDV and PDCoV demonstrate significant transboundary potential, the empirical evidence supporting specific dissemination routes is not equivalent between them. For PEDV, explosive global spread is backed by robust field and experimental data demonstrating an exceptionally low infectious dose, prolonged high‐titre faecal shedding, high stability under cold ambient temperatures, and well‐documented transmission via contaminated feed ingredients, transport vehicles and regional animal movement networks [3, 68, 71, 75, 123]. In contrast, while PDCoV shares similar potential transmission pathways, direct epidemiological evidence regarding its environmental persistence, infectious dose and large‐scale feedborne dissemination remains less extensive when compared to the massive empirical data available for PEDV [1, 42, 123].

Conversely, the apparent geographic restriction of emerging or recombinant entities such as SeCoV (confined to Europe) and SADS‐CoV (confined to Asia) should not be automatically interpreted as an intrinsically lower dissemination capacity [18, 19, 30, 33]. Rather, this localised footprint heavily reflects epidemiological confounders, including lower surveillance intensity, limited availability and implementation of routine differential diagnostics, varying reporting practices, and restricted sampling. This is underscored by the retrospective RT‐qPCR detection of SADS‐CoV in porcine samples from Vietnam, illustrating the threat of silent, undetected transboundary movement across Southeast Asia [34]. Ultimately, live breeding stock movements and globalised feed supply chains represent major universal vulnerabilities facilitating the transboundary emergence of both established and novel enteric CoVs [3, 4, 51].

### 9.2. Host Range, Interspecies Transmission and Zoonotic Risk

The receptor usage dynamics described previously directly govern the evolutionary potential and host range of these viruses. While the reliance on specific entry factors like APN or sialic acid restricts the tropism of viruses such as TGEV, PRCV and PDCoV [101, 120, 124], the exact functional dependency of PEDV and SeCoV remains disputed [7, 110, 116]. Furthermore, as previously detailed, SADS‐CoV stands out uniquely by circumventing standard CoV receptors altogether, relying instead on a highly conserved network of ubiquitously expressed host cofactors and proteases [32, 105, 110, 111]. This divergence in host–cell interactions directly dictates their interspecies transmission capacity, with scientific evidence ranging from strictly experimental observations to documented natural spillover events. TGEV and PRCV possess a well‐established capacity to infect and seroconvert companion animals (dogs and cats) under experimental and field settings, yet they pose no zoonotic risk [13, 14]. On the other end of the spectrum, PDCoV and SADS‐CoV possess the highest empirical and theoretical zoonotic potential. PDCoV has successfully crossed the species barrier under natural field conditions, with documented acute infections and viral isolation from children presenting with acute undifferentiated febrile illness in Haiti [29], alongside proven experimental pathogenicity in avian, bovine, rodent, and ferret models [25–28, 99]. Conversely, while SADS‐CoV has not yet been identified in human patients, its direct evolutionary lineage from bat CoVs (HKU2) [30, 32], its proven ability to experimentally infect diverse animal models, including rodents and avian species, its high efficiency in replicating within human respiratory and intestinal cell lines in vitro, and its proven ability to establish subclinical infections in murine models signal a potential zoonotic threat that demands rigorous public health surveillance [34, 36, 37, 105, 108].

### 9.3. Major Unresolved Questions for Surveillance and Control

A synthesis of current knowledge gaps reveals critical shared bottlenecks in the surveillance and management of porcine CoVs worldwide. First, the lack of commercially available, universally effective vaccines for newly emerging agents (such as SADS‐CoV, SeCoV and PDCoV) leaves global herds immunologically naïve and highly vulnerable to outbreaks [18, 22, 33]. For SADS‐CoV specifically, outbreak dynamics are further complicated by a localised and sporadic pattern that lacks clear temporal continuity, making re‐emergence hard to predict [34]. Second, the widespread field reliance on maternal immunity induction through planned feedback exposure (using autologous virulent material), while common for PEDV and TGEV, carries severe biosecurity risks, including the potential to inadvertently drive viral recombination and perpetuate endemic shedding [4, 21, 80]. For emerging pathogens like SADS‐CoV in particular, the use of controlled feedback is strongly discouraged; intentional exposure to uncharacterised intestinal material carries the extreme risk of amplifying the virus, propagating undetected co‐infections and providing the optimal environment for novel recombination events [34, 44, 80]. Lastly, active epidemiological surveillance is technically hindered by the lack of standardised, high‐throughput serological assays capable of differentiating between closely related alphacoronaviruses; specifically, the extensive cross‐reactivity between antibodies against TGEV, PRCV and the chimeric SeCoV, as well as potential cross‐reactivity with unidentified pathogens reported in SADS‐CoV serosurveys, represents a major roadblock for international trade and outbreak tracing [13, 18, 19, 103].

To address these vulnerabilities in the absence of universal vaccines, on‐the‐ground prevention must prioritise stringent, multi‐layered biosecurity protocols. Critical practical measures include the rigorous quarantine and testing of newly introduced stock to prevent the entry of asymptomatic or persistently shedding carrier animals. Furthermore, considering the documented risk of viral survival and transmission through contaminated feed ingredients and transport vehicles [123], the implementation of feed mitigation strategies, such as chemical treatments, extended holding times or thermal processing, and stringent equipment sanitisation [75], are essential interventions. Finally, to circumvent the limitations of serological cross‐reactivity and rapidly detect co‐infections or novel recombinants, routine herd monitoring must integrate comprehensive molecular diagnostic platforms, specifically multiplex reverse transcription‐PCR assays [70, 114]. These high‐throughput screening tools are indispensable for accurately differentiating enteric CoVs at the farm level, allowing for targeted and timely control measures before widespread transmission occurs.

### 9.4. Future Perspectives and Targeted Research Gaps

While the overarching challenge for swine enteric CoVs remains the strengthening of biosecurity and the development of universal diagnostics, targeted research must address the specific epidemiological gaps unique to each pathogen. For PEDV, future research should prioritise the development of next‐generation mucosal vaccines and defining the immunological basis of cross‐protection against rapidly evolving, recombination‐driven variants [42, 59]. Regarding PDCoV, studies must urgently clarify the molecular mechanisms facilitating its broad species tropism—specifically receptor usage and binding affinity across different hosts—to accurately assess its zoonotic spillover potential [25, 100]. For SADS‐CoV, priority must be given to ecological and epidemiological investigations aimed at uncovering the mechanisms behind its sporadic, temporally discontinuous emergence, as well as identifying its definitive intermediate hosts [34, 105]. For TGEV and PRCV, priorities must focus on refining differential serological assays to resolve cross‐reactivity that hinders trade, alongside monitoring potential TGEV re‐emergence through novel recombination events [13, 18]. Finally, for SeCoV and other potential chimaeras, the focus must be placed on genomic surveillance at the field level to map recombination hotspots among co‐circulating alphacoronaviruses and the development of highly specific diagnostic tools capable of resolving serological cross‐reactivity [18, 80].

## 10. Conclusions

Porcine enteric CoVs represent a persistent and evolving challenge for the global pig industry, characterised by their high transmissibility, capacity for interspecies transmission, and substantial economic impact. Among them, PEDV and TGEV remain the most historically significant pathogens, while emergent viruses such as SeCoV, PDCoV and SADS‐CoV illustrate the ongoing potential for viral recombination, adaptation, and the emergence of novel strains with enhanced virulence or an altered host range. Despite advances in biosecurity, molecular characterisation, diagnostics or even in vaccination, gaps persist in understanding the full epidemiological and pathogenic potential of these viruses, particularly for recombinants and newly emergent strains. Continuous surveillance, challenge studies and comparative pathogenesis research are therefore essential to anticipate future outbreaks, improve control strategies and mitigate the impact of swine enteric CoVs on both animal health and production systems.

## Author Contributions

Conceptualisation, bibliography search, writing – original draft: Héctor Puente. Conceptualisation and review: Héctor Arguello and Ana Carvajal. Review: Pedro Rubio.

## Funding

This work was funded by Spanish Ministerio de Ciencia e Innovación (Grant PID2024‐160714OB‐I00 and MICIU/AEI/10.13039/501100011033) and Junta de Castilla y Leon (Grant LE088P23).

## Disclosure

All authors have read and agreed to the published version of the manuscript.

## Conflicts of Interest

The authors declare no conflicts of interest.

## Data Availability

Data sharing not applicable to this article as no datasets were generated or analysed during the current study.
